# The Effect of Long-Term Particulate Matter Exposure on Respiratory Mortality: Cohort Study in China

**DOI:** 10.2196/56059

**Published:** 2024-09-24

**Authors:** Ying Wang, Zhuohao Wang, Jie Jiang, Tong Guo, Shimin Chen, Zhiqiang Li, Zhupei Yuan, Qiaoxuan Lin, Zhicheng Du, Jing Wei, Yuantao Hao, Wangjian Zhang

**Affiliations:** 1 Department of Medical Statistics, School of Public Health & Research Center for Health Information, Sun Yat-sen Global Health Institute, Sun Yat-sen University, 2nd Zhongshan Road, Guangzhou, 510000, China; 2 School of Public Health, Sun Yat-sen University, Guangzhou, China; 3 Department of Epidemiology and Biostatistics, School of Public Health, Peking University, Peking, China; 4 Department of Statistics, Guangzhou Health Technology Identification, Human Resources Assessment Center, Guangzhou, China; 5 Department of Atmospheric and Oceanic Science, Earth System Science Interdisciplinary Center, University of Maryland, College Park, MD, United States; 6 Peking University Center for Public Health and Epidemic Preparedness & Response, Key Laboratory of Epidemiology of Major Diseases (Peking University), Ministry of Education, Peking, China

**Keywords:** respiratory disease, mortality, particulate matter, causal inference, cohort study

## Abstract

**Background:**

Particulate matter (PM), which affects respiratory health, has been well documented; however, substantial evidence from large cohorts is still limited, particularly in highly polluted countries and for PM_1_.

**Objective:**

Our objective was to examine the potential causal links between long-term exposure to PMs (PM_2.5_, PM_10_, and more importantly, PM_1_) and respiratory mortality.

**Methods:**

A total of 580,757 participants from the Guangzhou area, China, were recruited from 2009 to 2015 and followed up through 2020. The annual average concentrations of PMs at a 1-km spatial resolution around the residential addresses were estimated using validated spatiotemporal models. The marginal structural Cox model was used to estimate the associations of PM exposure with respiratory mortality, accounting for time-varying PM exposure. Results were stratified by demographics and lifestyle behaviors factors.

**Results:**

Among the participants, the mean age was 48.33 (SD 17.55) years, and 275,676 (47.47%) of them were men. During the follow-up period, 7260 deaths occurred due to respiratory diseases. The annual average concentrations of PM_1_, PM_2.5_, and PM_10_ showed a declining trend during the follow-up period. After adjusting for confounders, a 6.6% (95% CI 5.6%‐7.6%), 4.2% (95% CI 3.6%‐4.7%), and 4.0% (95% CI 3.6%‐4.5%) increase in the risk of respiratory mortality was observed following each 1-μg/m^3^ increase in concentrations of PM_1_, PM_2.5_, and PM_10_, respectively. In addition, older participants, nonsmokers, participants with higher exercise frequency, and those exposed to a lower normalized difference vegetation index tended to be more susceptible to the effects of PMs. Furthermore, participants in the low-exposure group tended to be at a 7.6% and 2.7% greater risk of respiratory mortality following PM_1_ and PM_10_ exposure, respectively, compared to the entire cohort.

**Conclusions:**

This cohort study provides causal clues of the respiratory impact of long-term ambient PM exposure, indicating that PM reduction efforts may continuously benefit the population’s respiratory health.

## Introduction

The burden of chronic respiratory diseases remains severe, with nearly 545 million individuals, representing 7.4% of the world’s population, currently enduring a chronic respiratory condition. Chronic respiratory diseases were ranked as the third leading cause of death in 2017 [1]. Air pollution, particularly particulate matter (PM), has been recognized as 1 of the top 5 issues affecting health and the primary environmental predictor underlying the increased risk of respiratory diseases [2].

Existing evidence indicates that higher PM concentration significantly influenced respiratory health. A recent meta-analysis found that each 10-μg/m^3^ increment in PM_2.5_ and PM_10_ concentrations may increase the risk of death from respiratory diseases by 10% and 12%, respectively [3]. Quantifying the risk of respiratory mortality due to long-term PM exposure is crucial for developing effective long-term health-oriented strategies to reduce PM pollution.

However, existing studies are still facing several important challenges. First, a recent meta-analysis revealed the association between PM and respiratory mortality, in which most included studies were conducted in regions with low PM concentrations [3]. Only 5 cohort studies were from China, 3 of these only covered certain population groups (ie, men [4] and older people [5,6]), while the other 2 had a very limited number of outcome events (ie, n=72 [7] and n=67 [8]). Recently, although more investigations have examined the health impact of PM among Chinese residents [9-12], with most relying on ecological study designs or focused on short-term exposures, yet the availability of evidence from cohort studies or regarding long-term exposures remains limited.

Second, this limited number of existing studies predominantly examined the impacts of PM_2.5_ and PM_10_. Previous toxicological studies have revealed that the effect of PM may be significantly influenced by the particle size [13,14]. PM_1_, due to its smaller particle size and larger surface area, possesses greater penetration capabilities and higher toxicity compared to PM_2.5_ and PM_10_. However, the evidence assessing the effects of PM_1_ exposure on respiratory health is scarce. Additionally, existing studies are generally based on traditional association assessments such as logistic and Cox models, which assess the impact of confounding by comparing adjusted and crude estimates; however, this strategy is flawed due to the noncollapsible effect (ie, the effect estimates change upon including a certain covariate in the model, even if the covariate is unrelated to the outcome) [15]. Causal inference approaches were recently developed for the observational data, with the specific advantage of simulating randomized controlled trials, ensuring the exchangeability of the exposed and unexposed populations, and enabling effect estimates to be specifically attributed to a certain exposure [16]. However, such causal clues are quite limited in existing studies.

In this study, our objective was to examine the association between long-term exposure to PMs (PM_2.5_, PM_10_, and more importantly, PM_1_) and respiratory mortality among Chinese adults based on the causal inference framework, and then potential modifiers of these associations were also explored.

## Methods

### Study Design and Population

Although it is a part of the community-based collaborative innovation program against the hepatitis B virus, the cohort was established to investigate the prevalence of, as well as the underlying predictors for, several major infectious diseases, such as hepatitis B virus, and major chronic diseases, such as respiratory and cardiovascular outcomes [17]. Due to the availability of data, this study recruited 654,115 participants from 35 randomly selected communities in Guangzhou between 2009 and 2015 [18,19]. Inclusion criteria included participants being permanent residents, capable of undergoing a physical examination, and willing to participate with a signed informed consent form. Exclusion criteria included an inability to complete the questionnaire or physical examination, or the presence of significant mental or cognitive abnormalities evaluated through a combination of past medical history and subjective verbal reports. The flowchart of the study sample selection can be found in Figure S1 in Multimedia Appendix 1. Participants younger than 18 years (n=72,330) or with unknown cause of death (n=1028) were excluded, resulting in a total of 580,757 participants who followed up till 2020.

The information was collected by the interviewers using a computer-based standardized structured questionnaire, which included general demographic characteristics, lifestyle behaviors, and other variables. Physical activity was assessed by participants’ self-reported frequency of exercise in the past week. BMI (kg/m^2^) was calculated by dividing weight by the square of height, with height and weight being measured according to standard protocols.

### Ethical Considerations

The data in our study has been de-identified. This study, which did not involve invasive measures on the participants, was thoroughly reviewed and approved by the institutional review board committee at Sun Yat-sen University (L2017030), ensuring all procedures followed the relevant ethical tenets of the Declaration of Helsinki.

### Outcome Definition

We determined the vital status of each participant during the period from enrollment to December 2020 and confirmed the cause of death for those who died by linking the cohort data to the Death Registry Systems of the Guangzhou Centers for Disease Control and Prevention via the national ID. The main outcome of this study was mortality from respiratory diseases (*International Classification of Diseases, 10th Revision* [ICD-10]: J00-J99).

### Exposure Assessment

Ambient PM data at a 1-km spatial resolution over mainland China were extracted from the ChinaHighAirPollutants database, which has been used in prior studies [20,21]. The concentrations of PM_1_, PM_2.5_, and PM_10_, which are more likely to be objective, were obtained from space-time extremely randomized tree models based on meteorological, land use information, and other factors [22-25]. Initially, we converted the residential addresses of participants into standardized longitude and latitude coordinates, and assigned the annual average levels of PMs based on those coordinates using a nearest-neighbor matching approach.

The normalized difference vegetation index (NDVI) is an objective indicator of vegetation cover provided by the Terra Moderate Resolution Imaging Spectroradiometer (MODIS) Vegetation Indices (MOD13Q1). The average NDVI value within a 500-meter buffer around each person’s residential address was assigned based on latitude and longitude coordinates.

### Statistical Analysis

The marginal structural Cox model was used to estimate the associations of PM exposure with respiratory mortality, accounting for time-varying variables in the model. This approach is a causal inference method for observational data, which have been increasingly used in recent years [26]. First, we calculated the generalized propensity scores by regressing the exposure variable against the observed confounders. Stabilized inverse probability weights (IPWs) were then developed by using the inverse of the generalized propensity scores to weigh each participant (ie, creating a pseudopopulation) [27]. Then, a time-varying Cox model was developed to estimate the associations of PM exposure with respiratory mortality, accounting for time-varying variables in the model. A total of 3 different methods (ie, linear model, generalized estimating equation, and machine learning) were used to create the IPW, with the generalized estimating equation weighting method being selected due to its superior performance in covariates balancing as diagnosed by the minimal average absolute correlation values (Figure S2 in Multimedia Appendix 1). The generalized variance-inflation factor was used to assess collinearity among the covariates, with values less than 2 indicating no issues of collinearity. Additional methods’ details can be found elsewhere [28].

Table S1 in Multimedia Appendix 1 shows the proportion of missing data for all variables, which were imputed using the chained equation [29]. The complete data set after imputation was used in this study. Finally, we developed 3 models.

First, model 0 was an unadjusted model within the time-varying Cox model framework. Second, model 1 was model 0 plus confounders, including sex, age, demographic and behavioral factors, and NDVI, which were selected based on prior studies. Finally, model 2 was this model refitting model 1 using the marginal structural Cox model and was the final model. Subsequently, the results from the ultimate models were stratified. To investigate the potential influence of exposure to low concentrations, the analysis was further restricted to individuals having an annual PM_10_ (the largest particles containing PM_2.5_ and PM_1_) concentration below 70 μg/m^3^ (World Health Organization [WHO] interim target 1) [30].

Several sensitivity analyses were carried out. First, we compared the main results using 3 different IPW methods. Second, the analysis was repeated using nonimputed data to assess whether the results were influenced by the imputation of missing data. Third, we used different sizes of buffers for the NDVI (ie, 250 m and 1000 m), and assessed the potential impact of unmeasured confounding factors in observational studies using the E-value [31].

## Results

**Figure 1.**
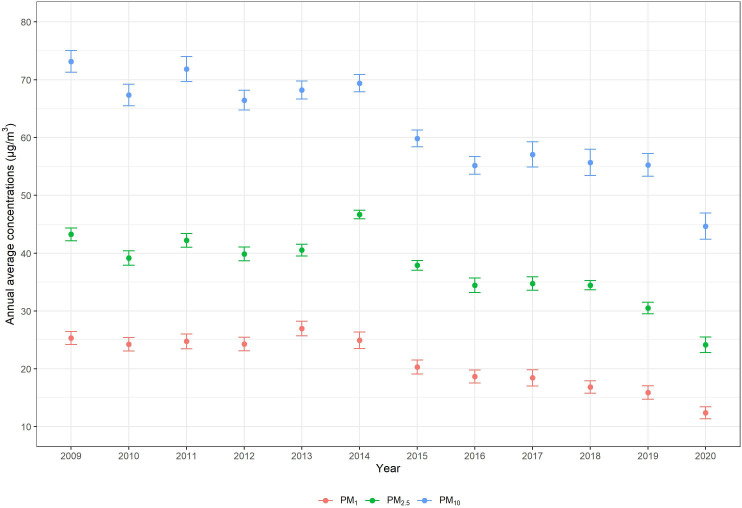
The distribution of annual average concentrations of particulate matter during 2009 and 2020 in Guangzhou. PM_1_: particulate matter with an aerodynamic diameter ≤1 μm; PM_2.5_: particulate matter with an aerodynamic diameter ≤2.5 μm; PM_10_: particulate matter with an aerodynamic diameter ≤10 μm.

Of the 580,757 participants, the mean age was 48.33 (SD 17.55) years, and 275,676 (47.47%) of them were men (Table 1). There were 7260 deaths from respiratory diseases during the average 8 years of follow-up. Participants who died from respiratory diseases tended to be older (78.01 years vs 47.95 years), men (4173/7260, 57.48% vs 271,503/573,497, 47.34%), of Han ethnicity (7229/7260, 99.57% vs 562,575/573,497, 98.10%), widowed (1257/7260, 17.31% vs 16,005/573,497, 2.79%), or have lower education (illiterate or semiliterate: 496/7260, 6.83% vs 6107/573,497; all *P*<.001) compared to others. Moreover, they tended to be current smokers (909/7260, 12.52% vs 67,747/573,497, 11.81%), had a higher level of exercise frequency (1598/7260, 22.01% vs 114,996/573,497, 20.05%), and had lower NDVI exposure (0.207 vs 0.214; all *P*<.001). Figure 1 shows the annual average concentrations of PMs declined during the follow-up period.

**Figure 2.**
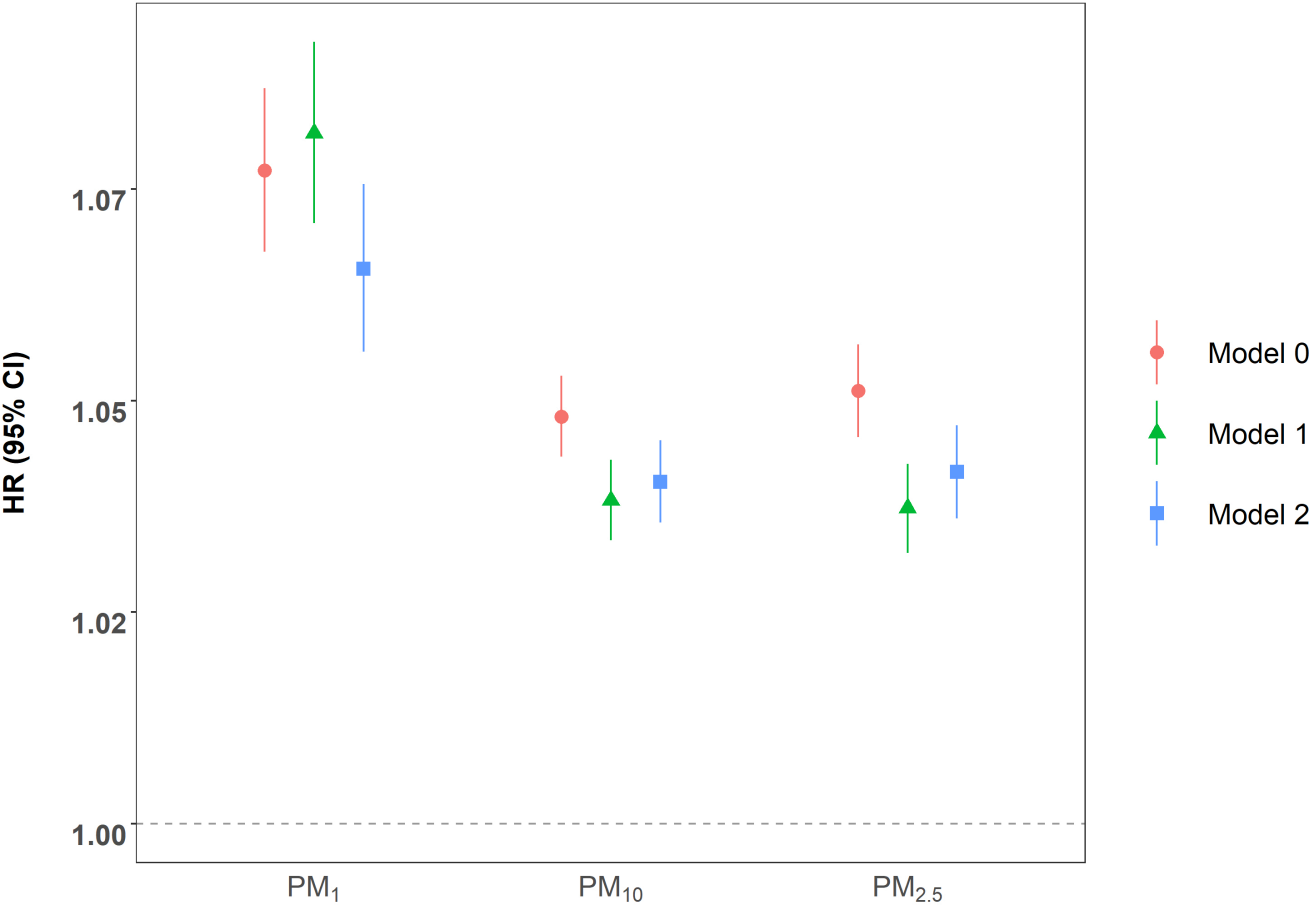
Association between 1-μg/m^3^ increase in long-term particulate matter exposure and respiratory mortality. Model 0 was a crude model under a time-varying Cox model. Model 1 was the model additionally adjusted for sex, age, ethnicity, education level, marital status, smoking status, medical insurance, exercise frequency, and NDVI (500 m) based on model 0. Model 2 was the marginal structural Cox model based on model 1. HR: hazard ratio; NDVI: normalized difference vegetation index; PM_1_: PM with an aerodynamic diameter ≤1 μm; PM_2.5_: PM with an aerodynamic diameter ≤2.5 μm; PM_10_: PM with an aerodynamic diameter ≤10 μm.

Figure 2 shows the associations of PM exposure with respiratory mortality. In the main model, a 1-μg/m^3^ increase in PM_1_, PM_2.5_, and PM_10_ concentrations corresponded to increases in the risk of respiratory mortality by 6.6% (95% CI 5.6%‐7.6%), 4.2% (95% CI 3.6%‐4.7%), and 4.0% (95% CI 3.6%‐4.5%), respectively. Simultaneously, the effect estimates were similar when using adjusted traditional Cox models.

In stratified analyses (Table 2), a significantly higher effect of the PM_1_-respiratory mortality association was observed among individuals aged 65 years and older (hazard ratio [HR] 1.096, 95% CI 1.086‐1.107) compared to their younger counterparts (HR 1.067, 95% CI 1.036‐1.100). A similar interaction effect was observed for PM_2.5_ and PM_10_ in association with respiratory mortality. Compared with participants who never smoked (HR ranging 1.043‐1.071 across PMs), those who had ever smoked (HR ranging 1.006‐1.019) or currently smoked (HR ranging 1.021‐1.038) generally had a lower risk of respiratory mortality following PMs exposures. Significant modification effects were also observed for exercise frequency, although the inter-strata disparity was inconsistent across different sizes of PMs. Participants with high exercise frequency (HR ranging 1.046‐1.073 across PMs) experienced a greater effect estimate than those with low exercise frequency (HR ranging 1.032‐1.068 across PMs). Meanwhile, individuals in the low exercise frequency group showed a higher effect of PM_1_ and PM_10_ on respiratory mortality compared to those with moderate exercise frequency but exhibited a lower risk related to PM_2.5_ exposure. Furthermore, the associations appeared to be greater among participants with lower NDVI exposure compared to those with higher NDVI levels. No significant differences in PM-respiratory mortality associations were observed across groups by sex, marital status, or education level.

**Table 1.** Baseline characteristics of the cohort participants enrolled during 2009 and 2015 in Guangzhou.

Characteristic	Overall	Overall respiratory deaths	Control group	*P* value
Number of participants, n	580,757	7260	573,497	—^a^
Age (years), mean (SD)	48.33 (17.55)	78.01 (10.41)	47.95 (17.29)	<.001
Sex (men), n (%)	275,676 (47.47)	4173 (57.48)	271,503 (47.34)	<.001
Ethnic (minority), n (%)	10,953 (1.89)	31 (0.43)	10,922 (1.90)	<.001
**Education, n (%)**				<.001
Illiterate or semiliterate	6603 (1.14)	496 (6.83)	6107 (1.06)	
Primary school	67,247 (11.58)	2464 (33.94)	64,783 (11.30)	
Second school	131,394 (22.62)	1796 (24.74)	129,598 (22.60)	
High school	266,467 (45.88)	2167 (29.85)	264,300 (46.09)	
College or above	109,046 (18.78)	337 (4.64)	108,709 (18.96)	
**Marital status, n (%)**				<.001
Never married	108,424 (18.67)	484 (6.67)	107,940 (18.82)	
Married	448,138 (77.16)	5423 (74.70)	442,715 (77.20)	
Widowed	17,262 (2.97)	1257 (17.31)	16,005 (2.79)	
Divorce	6933 (1.20)	96 (1.32)	6837 (1.19)	
**Medical insurance, n (%)**				<.001
Medical insurance for urban workers	354,396 (61.02)	4507 (62.08)	349,889 (61.01)	
Medical insurance for urban residents	164,213 (28.28)	2385 (32.85)	161,828 (28.22)	
The new rural cooperative medical insurance	6439 (1.11)	17 (0.23)	6422 (1.12)	
Others	55,709 (9.59)	351 (4.83)	55,358 (9.65)	
BMI (kg/m^2^), mean (SD)	22.07 (2.45)	21.99 (2.67)	22.07 (2.45)	.14
**Smoking status, n (%)**				<.001
Never	505,385 (87.02)	6033 (83.10)	499,352 (87.07)	
Ever	6716 (1.16)	318 (4.38)	6398 (1.12)	
Current	68,656 (11.82)	909 (12.52)	67,747 (11.81)	
**Alcohol consumption, n (%)**				.98
Never	515,205 (88.71)	6440 (88.71)	508,765 (88.71)	
Ever	65,552 (11.29)	820 (11.29)	64,732 (11.29)	
**Exercise frequency, n (%)**				<.001
Low	322,225 (55.48)	3965 (54.61)	318,260 (55.49)	
Moderate	141,938 (24.44)	1697 (23.38)	140,241 (24.45)	
High	116,594 (20.08)	1598 (22.01)	114,996 (20.05)	
NDVI^b^ (500 m), mean (SD)	0.213 (0.040)	0.207 (0.037)	0.214 (0.040)	<.001

a Not applicable.

b NDVI: normalized difference vegetation index.

**Table 2.** The modification effect of basic characteristics and behavior factors on the association between particulate matter and respiratory mortality.

	PM_1_^a^		PM_2.5_^b^		PM_10_^c^	
Effect modifiers	HR^d^ (95% CI)	*P* value	HR (95% CI)	*P* value	HR (95% CI)	*P* value
**Age (years)**						
<65	1.067 (1.036‐1.100)	Reference	1.035 (1.018‐1.052)	Reference	1.031 (1.016‐1.046)	Reference
≥65	1.096 (1.086‐1.107)	<.001	1.049 (1.044‐1.054)	<.001	1.049 (1.044‐1.054)	<.001
**Gender**						
Men	1.048 (1.035‐1.061)	Reference	1.034 (1.027‐1.041)	Reference	1.034 (1.028‐1.041)	Reference
Women	1.086 (1.071‐1.102)	.99	1.050 (1.042‐1.059)	.71	1.047 (1.040‐1.054)	.98
**Education**						
Primary school and below	1.061 (1.046‐1.076)	Reference	1.044 (1.035‐1.052)	Reference	1.045 (1.037‐1.052)	Reference
Second and high school	1.029 (1.016‐1.042)	.88	1.031 (1.024‐1.038)	.61	1.026 (1.020‐1.032)	.93
College or above	1.069 (1.024‐1.117)	.08	1.041 (1.016‐1.066)	.16	1.039 (1.018‐1.061)	.09
**Marital status**						
Never married	1.068 (1.025‐1.113)	Reference	1.043 (1.019‐1.068)	Reference	1.046 (1.024‐1.069)	Reference
Married	1.067 (1.056‐1.079)	.74	1.040 (1.034‐1.046)	.82	1.039 (1.034‐1.044)	.95
Widowed or divorce	1.073 (1.050‐1.096)	.44	1.042 (1.030‐1.054)	.75	1.039 (1.029‐1.050)	.58
**Smoke status**						
Never	1.071 (1.060‐1.082)	Reference	1.046 (1.040‐1.052)	Reference	1.043 (1.037‐1.048)	Reference
Ever	1.019 (0.975‐1.065)	.02	1.006 (0.985‐1.028)	.02	1.015 (0.996‐1.036)	.05
Current	1.038 (1.011‐1.066)	.001	1.021 (1.007‐1.035)	<.001	1.024 (1.012‐1.036)	<.001
**Exercise frequency**						
Low	1.068 (1.048‐1.089)	Reference	1.032 (1.022‐1.042)	Reference	1.036 (1.027‐1.045)	Reference
Moderate	1.048 (1.028‐1.068)	<.001	1.037 (1.026‐1.048)	.001	1.035 (1.025‐1.045)	.001
High	1.073 (1.060‐1.087)	.045	1.050 (1.042‐1.058)	.03	1.046 (1.039‐1.053)	.04
**NDVI** ^e^						
First tertile	1.049 (1.033‐1.065)	Reference	1.047 (1.038‐1.055)	Reference	1.039 (1.031‐1.047)	Reference
Second tertile	0.983 (0.967‐0.999)	<.001	1.013 (1.004‐1.023)	<.001	1.012 (1.004‐1.020)	<.001
Third tertile	1.041 (1.021‐1.062)	<.001	1.018 (1.007‐1.029)	<.001	1.024 (1.015‐1.033)	<.001

a PM_1_: particulate matter with an aerodynamic diameter ≤1 μm.

b PM_2.5_: particulate matter with an aerodynamic diameter ≤2.5 μm.

c PM_10_: particulate matter with an aerodynamic diameter ≤10 μm.

d HR: hazard ratio.

e NDVI: normalized difference vegetation index.

The results also revealed that PM exposure was linked to respiratory mortality in the low-exposure group (Figures S3 and S4 in Multimedia Appendix 1). For the low-exposure group, each 1-μg/m³ increase in PM_10_ and PM_1_ concentrations corresponded to a 6.7% and 14.2% increase in respiratory mortality. In contrast, the estimates for the entire cohort were 4.0% and 6.6% for PM_10_ and PM_1_, respectively, resulting in a 2.7% and 7.6% greater risk among the low-exposure group.

In sensitivity analyses, the results from 3 different IPW methods, or the model excluding participants with missing values, or using the other 2 different NDVI buffers were similar to the main models (Table S2 in Multimedia Appendix 1). The E-values also indicate that the results were robust (Table S3 in Multimedia Appendix 1).

## Discussion

### Principal Findings

In a Chinese community-based cohort study, our results showed that each 1-μg/m^3^ increase in the concentration of PM_1_, PM_2.5_, and PM_10_ was linked to increases in the risk of respiratory mortality by 6.6%, 4.2%, and 4.0%, respectively. Moreover, this risk was reported to be higher among older participants, never-smokers, participants with high exercise frequency, and those residing in low-NDVI areas than among their counterparts. Participants in the low-exposure group tended to be at a 7.6% and 2.7% greater risk of respiratory mortality following PM_1_ and PM_10_ exposure, respectively, compared to the entire cohort.

### Effect of Long-Term PM Exposure

Our findings showed significant associations of PM exposure with respiratory mortality. Former research on the PM_2.5_-respiratory mortality association yielded inconclusive results; a 2013 meta-analysis found a nonsignificant association [32]. However, another updated meta-analysis of 17 studies indicated an elevated risk of respiratory mortality attributable to PM_2.5_ and PM_10_ [3]. In recent years, an increasing number of studies have focused on the health impact of PM_1_. A time-series study in China examined the effects of short-term exposure to PM on mortality, showing that a 10-μg/m^3^ increase in PM_1_ concentration was associated with a 0.35% increase in the excess risk of daily mortality [33]. Another 2 case-crossover studies in China found the same increase was linked to a 9% and 56% increase in the hospitalization risk of respiratory diseases and the incidence of doctor-diagnosed asthma, respectively [34,35]. Our research provides further insights into the establishment of national standards concerning PM_1_ and developing policies to reduce the health risks associated with ambient air pollution. In the future, it is essential to explore the biological mechanisms involved, including how PM penetrates respiratory systems and the biochemical interactions at the cellular level.

### Susceptible Populations

Stratified analysis showed that the older participants and never-smokers were more susceptible to the respiratory impact of PMs than their counterparts. A systematic review of 20 studies consistently indicated greater vulnerability among older adults [36]. One possible explanation is that aging reduces the physiological capacity in the lungs for gas exchange, respiratory mechanics, muscle strength, and ventilatory control, causing prolonged exposure to toxins and resulting in greater lung damage [37]. In regard to the modification effect of smoking status, a cross-sectional study across 6 countries consistently found that the PM_2.5_-stroke effect among never-smokers was greater than that among ever-smokers [38]. However, the exact mechanisms need to be further investigated. The mechanisms underlying the interaction between smoking and PMs remain unclear. One possible explanation is that both smoking and exposure to ambient PMs have negative effects on health through pathways involving oxidative stress and inflammation [39]. It also suggests that the negative effects of smoking are dominant in smokers due to their limited kidney function reserve [40], so additional exposure to ambient PMs may not further enhance the effects. However, further research on mechanisms is needed to clarify this hypothesis.

Additionally, we observed that participants with high exercise frequency and lower green space exposure showed higher vulnerability to PMs. The interaction effects between physical activity and PM exposure are controversial in prior studies. In a meta-analysis, exposure to exercise-related air pollution may reduce the positive effects of physical activity on cerebral health [41]. A recent cohort study conducted in China found that when the PM_2.5_ concentrations exceeded 54 μg/m^3^, participants with farming activity levels greater than 15.74 metabolic equivalent of task (MET)–hours per day had a 12% and 18% elevated risk of cerebrovascular disease compared to those with activity levels between 3.43‐8.05 and 8.06‐15.73 MET-hours per day, respectively [42]. Another cohort study across 6 countries revealed that high levels of physical activity could increase the association between PM and stroke [43]. To further clarify this hypothesis, more research is required. Regarding the interactions between PM and green space exposure, in line with our findings, a cohort study among Chinese populations indicated a synergistic effect on mortality when PM decreased while greenness increased [44]. Possible reasons for the health benefits of green space exposure include that green plants absorb harmful air pollutants and help alleviate mental stress, reducing symptoms of anxiety and depression, and promoting psychological relaxation [45]. Therefore, both existing evidence and our findings suggest that greenness can serve as a protective measure to mitigate the adverse effects of air pollution.

Another significant finding was that the low-exposure group showed a stronger association between respiratory mortality and PM exposure compared to the overall population. Our results align with other research that has also found a greater association between PM and mortality among those exposed to lower air pollution concentrations [46,47]. A cohort study among the Canadians showed a 10.4% greater effect estimate over the 0‐5 μg/m^3^ range compared to the 5‐12 μg/m^3^ range (23.7% vs 13.3%) [48]. A possible reason is that participants in highly polluted areas may take measures to protect themselves from the harmful impacts of ambient air pollutants on health, such as turning on air purifiers or wearing masks during severe air pollution days. These results suggest that the same concentration reduction in air pollution at relatively lower levels may result in greater health improvements.

### Strengths and Limitations

We provided the first cohort evidence of the impact of long-term ambient PM exposure across different particle sizes (especially PM_1_) on respiratory mortality among the general Chinese population. Currently, there are an insufficient number of epidemiological studies on the effects of PM_1_, and no established monitoring standards exist for long-term PM_1_ exposure. Therefore, our study provides important quantitative evidence for the establishment of standards and guidelines for PM_1_. In addition, our study used causal inference models and considered the temporal changes in PM features by using time-varying PM data, which ensured more robust effect estimates.

However, some limitations need to be discussed. First, the environmental exposure data for the study participants were obtained by matching the pollution simulation with each participant’s residential address. Other individual factors, such as personal indoor air pollution and outdoor physical activity time, were not considered, which may induce individual exposure measurement errors. However, this type of exposure misclassification is usually considered nondifferential and not likely to result in significant bias [49]. Second, owing to the absence of specific sources or chemical components of air pollution, our study could not identify the specific chemical components contributing to the higher risk of mortality from respiratory disease. Third, although we adjusted for demographic characteristics, socioeconomic status, lifestyle factors, and other potential confounding variables, residual confounding could not be ruled out due to the presence of unobserved confounding factors, such as occupational exposure to air pollutants or indoor air quality. However, the sensitivity analysis of E-values indicated that the effect estimates were robust against unobserved confounding factors.

### Conclusions

This study suggests potential causal links between PM exposure and respiratory mortality. In addition, older participants, nonsmokers, participants with high exercise frequency, and low-NDVI residents, as well as those in the low-exposure group, appear to exhibit a higher vulnerability to the impacts of PMs, highlighting the necessity for developing strategies focused on reducing PM concentrations and protecting susceptible populations.

## Supplementary material

10.2196/56059 Multimedia Appendix 1 The flowchart of the study sample selection.

## Abbreviations

HR: hazard ratio
ICD-10: *International Classification of Diseases, 10th Revision*
IPW: inverse probability weight
MET: metabolic equivalent of task
MODIS: Moderate Resolution Imaging Spectroradiometer
NDVI: normalized difference vegetation index
PM: particulate matter
WHO: World Health Organization

## References

[1] GBD Chronic Respiratory Disease Collaborators (2020). Prevalence and attributable health burden of chronic respiratory diseases, 1990-2017: a systematic analysis for the Global Burden of Disease Study 2017. Lancet Respir Med.

[2] Zhou M, Wang H, Zeng X (2019). Mortality, morbidity, and risk factors in China and its provinces, 1990-2017: a systematic analysis for the Global Burden of Disease Study 2017. Lancet.

[3] Chen J, Hoek G (2020). Long-term exposure to PM and all-cause and cause-specific mortality: a systematic review and meta-analysis. Environ Int.

[4] Zhou M, Liu Y, Wang L, Kuang X, Xu X, Kan H (2014). Particulate air pollution and mortality in a cohort of Chinese men. Environ Pollut.

[5] Wong CM, Lai HK, Tsang H (2015). Satellite-based estimates of long-term exposure to fine particles and association with mortality in elderly Hong Kong residents. Environ Health Perspect.

[6] Yang Y, Tang R, Qiu H (2018). Long term exposure to air pollution and mortality in an elderly cohort in Hong Kong. Environ Int.

[7] Dong GH, Zhang P, Sun B (2012). Long-term exposure to ambient air pollution and respiratory disease mortality in Shenyang, China: a 12-year population-based retrospective cohort study. Respiration.

[8] Chen X, Wang X, Huang J ju (2017). Nonmalignant respiratory mortality and long-term exposure to PM10 and SO2: a 12-year cohort study in Northern China. Environ Pollut.

[9] Liu W, Wei J, Cai M (2022). Particulate matter pollution and asthma mortality in China: a nationwide time-stratified case-crossover study from 2015 to 2020. Chemosphere.

[10] Liu C, Cai J, Chen R (2022). Coarse particulate air pollution and daily mortality: a global study in 205 cities. Am J Respir Crit Care Med.

[11] Yin P, Brauer M, Cohen AJ (2020). The effect of air pollution on deaths, disease burden, and life expectancy across China and its provinces, 1990-2017: an analysis for the Global Burden of Disease Study 2017. Lancet Planet Health.

[12] Qin G, Wang X, Wang T (2022). Impact of particulate matter on hospitalizations for respiratory diseases and related economic losses in Wuhan, China. Front Public Health.

[13] Yang M, Zeng HX, Wang XF (2023). Sources, chemical components, and toxicological responses of size segregated urban air PM samples in high air pollution season in Guangzhou, China. Sci Total Environ.

[14] Yang M, Jalava P, Hakkarainen H (2022). Fine and ultrafine airborne PM influence inflammation response of young adults and toxicological responses in vitro. Sci Total Environ.

[15] Pang M, Kaufman JS, Platt RW (2016). Studying noncollapsibility of the odds ratio with marginal structural and logistic regression models. Stat Methods Med Res.

[16] Santacatterina M, García-Pareja C, Bellocco R, Sönnerborg A, Ekström AM, Bottai M (2019). Optimal probability weights for estimating causal effects of time-varying treatments with marginal structural Cox models. Stat Med.

[17] Ruan B, Yu Z, Yang S (2019). Establishment and development of national community-based collaborative innovation demonstration areas to achieve the control target of hepatitis B in China. BMC Infect Dis.

[18] Guo T, Chen S, Wang Y (2024). Potential causal links of long-term air pollution with lung cancer incidence: from the perspectives of mortality and hospital admission in a large cohort study in Southern China. Int J Cancer.

[19] Zhang Y, Wang Y, Du Z (2023). Potential causal links between long-term ambient particulate matter exposure and cardiovascular mortality: new evidence from a large community-based cohort in South China. Ecotoxicol Environ Saf.

[20] Xu H, Guo B, Qian W (2021). Dietary pattern and long-term effects of particulate matter on blood pressure: a large cross-sectional study in Chinese adults. Hypertension.

[21] Yu Y, Lin H, Liu Q (2024). Association of residential greenness, air pollution with adverse birth outcomes: results from 61,762 mother‑neonatal pairs in project ELEFANT (2011–2021). Sci Total Environ.

[22] Wei J, Huang W, Li Z (2019). Estimating 1-km-resolution PM2.5 concentrations across China using the space-time random forest approach. Remote Sens Environ.

[23] Wei J, Li Z, Guo J (2019). Satellite-derived 1-km-resolution PM1 concentrations from 2014 to 2018 across China. Environ Sci Technol.

[24] Wei J, Li Z, Lyapustin A (2021). Reconstructing 1-km-resolution high-quality PM2.5 data records from 2000 to 2018 in China: spatiotemporal variations and policy implications. Remote Sens Environ.

[25] Wei J, Li Z, Xue W (2021). The ChinaHighPM10 dataset: generation, validation, and spatiotemporal variations from 2015 to 2019 across China. Environ Int.

[26] Robins JM, Hernán MA, Brumback B (2000). Marginal structural models and causal inference in epidemiology. Epidemiology.

[27] Cole SR, Hernan MA (2008). Constructing inverse probability weights for marginal structural models. Am J Epidemiol.

[28] Wang Y, Du Z, Zhang Y (2023). Long-term exposure to particulate matter and COPD mortality: insights from causal inference methods based on a large population cohort in Southern China. Sci Total Environ.

[29] van Buuren S, Groothuis-Oudshoorn K (2011). mice: multivariate imputation by chained equations in R. J Stat Softw.

[30] World Health Organization (2021). WHO Global Air Quality Guidelines: Particulate Matter (PM2.5 and PM10), Ozone, Nitrogen Dioxide, Sulfur Dioxide and Carbon Monoxide.

[31] Haneuse S, VanderWeele TJ, Arterburn D (2019). Using the E-value to assess the potential effect of unmeasured confounding in observational studies. JAMA.

[32] Hoek G, Krishnan RM, Beelen R (2013). Long-term air pollution exposure and cardio-respiratory mortality: a review. Environ Health.

[33] Wang H, Yin P, Fan W (2021). Mortality risk associated with short-term exposure to particulate matter in China: estimating error and implication. Environ Sci Technol.

[34] Yang M, Chu C, Bloom MS (2018). Is smaller worse? New insights about associations of PM1 and respiratory health in children and adolescents. Environ Int.

[35] Zhang Y, Ding Z, Xiang Q, Wang W, Huang L, Mao F (2020). Short-term effects of ambient PM1 and PM2.5 air pollution on hospital admission for respiratory diseases: case-crossover evidence from Shenzhen, China. Int J Hyg Environ Health.

[36] Sacks JD, Stanek LW, Luben TJ (2011). Particulate matter-induced health effects: who is susceptible?. Environ Health Perspect.

[37] Vaz Fragoso CA, Gill TM (2012). Respiratory impairment and the aging lung: a novel paradigm for assessing pulmonary function. J Gerontol A Biol Sci Med Sci.

[38] Ai S, Qian ZM, Guo Y (2019). Long-term exposure to ambient fine particles associated with asthma: a cross-sectional study among older adults in six low- and middle-income countries. Environ Res.

[39] Zhang Z, Guo C, Lau AKH (2018). Long-term exposure to fine particulate matter, blood pressure, and incident hypertension in Taiwanese adults. Environ Health Perspect.

[40] Tang YX, Zhang YT, Xu YJ (2023). Exposure to ambient particulate matter and hyperuricemia: an eight-year prospective cohort study on male traffic officers in China. Ecotoxicol Environ Saf.

[41] Bos I, De Boever P, Int Panis L, Meeusen R (2014). Physical activity, air pollution and the brain. Sports Med.

[42] Sun D, Liu C, Ding Y (2023). Long-term exposure to ambient PM2·5, active commuting, and farming activity and cardiovascular disease risk in adults in China: a prospective cohort study. Lancet Planet Health.

[43] Lin H, Guo Y, Di Q (2017). Ambient PM2.5 and stroke: effect modifiers and population attributable risk in six low- and middle-income countries. Stroke.

[44] Ji JS, Zhu A, Lv Y, Shi X (2020). Interaction between residential greenness and air pollution mortality: analysis of the Chinese Longitudinal Healthy Longevity Survey. Lancet Planet Health.

[45] Nieuwenhuijsen MJ, Khreis H, Triguero-Mas M, Gascon M, Dadvand P (2017). Fifty shades of green: pathway to healthy urban living. Epidemiology.

[46] Wu X, Braun D, Schwartz J, Kioumourtzoglou MA, Dominici F (2020). Evaluating the impact of long-term exposure to fine particulate matter on mortality among the elderly. Sci Adv.

[47] Alexeeff SE, Deosaransingh K, Liao NS, Van Den Eeden SK, Schwartz J, Sidney S (2021). Particulate matter and cardiovascular risk in adults with chronic obstructive pulmonary disease. Am J Respir Crit Care Med.

[48] Pinault LL, Weichenthal S, Crouse DL (2017). Associations between fine particulate matter and mortality in the 2001 Canadian Census Health and Environment Cohort. Environ Res.

[49] Zeger SL, Thomas D, Dominici F (2000). Exposure measurement error in time-series studies of air pollution: concepts and consequences. Environ Health Perspect.

