# Supplementary material for: The Effect of Long-Term Particulate Matter Exposure on Respiratory Mortality: Cohort Study in China
Source: JMIR Public Health Surveill. 2024 Sep 24;10:e56059. doi: 10.2196/56059 (PMC11444524; doi:10.2196/56059)
Supplement: Multimedia Appendix 1 [file publichealth-v10-e56059-s001.docx]

| Table S1. Summary statistics and baseline characteristics of the study subjects | | | |
| --- | --- | --- | --- |
| Characteristics | Total | Complete cases | Missing |
|  | (N=580,757) | (N=380,567) | n (%) |
| Demographics |  |  |  |
| Age (mean (SD), years) | 48.33 (17.55) | 48.25 (17.16) | 0 (0) |
| Sex, (men %) | 275676 (47.5) | 177606 (46.7) | 0 (0) |
| Ethnic, (minority, %) | 10953 (1.9) | 2453 (0.6) | 593 (0.10) |
| Education (%) |  |  | 102524 (17.65) |
| Illiterate or semiliterate | 6603 (1.1) | 5473 (1.4) |  |
| Primary school | 67247 (11.6) | 42101 (11.1) |  |
| Second school | 131394 (22.6) | 102628 (27.0) |  |
| High school | 266467 (45.9) | 146937 (38.6) |  |
| College or above | 109046 (18.8) | 83428 (21.9) |  |
| Marital status (%) |  |  | 61885 (10.66) |
| Never married | 108424 (18.7) | 67899 (17.8) |  |
| Married | 448138 (77.1) | 294806 (77.5) |  |
| Widowed | 17262 (3.0) | 12877 (3.4) |  |
| Divorce | 6933 (1.2) | 4985 (1.3) |  |
| Medical insurance (%) |  |  | 42103 (7.25) |
| Medical insurance for urban workers | 354396 (61.0) | 234028 (61.5) |  |
| Medical insurance for urban residents | 164213 (28.3) | 100273 (26.3) |  |
| The new rural cooperative medical insurance | 6439 (1.1) | 5064 (1.3) |  |
| Others | 55709 (9.6) | 41202 (10.8) |  |
| Smoking status (%) |  |  | 39084 (6.73) |
| Never | 505385 (87.0) | 350468 (92.1) |  |
| Ever | 6716 (1.2) | 5320 (1.4) |  |
| Current | 68656 (11.8) | 24779 (6.5) |  |
| Alcohol consumption (%) |  |  | 39426 (6.79) |
| Never | 515205 (88.7) | 359566 (94.5) |  |
| Ever | 65552 (11.3) | 21001 (5.5) |  |
| Exercise frequency (%) |  |  | 39003 (6.72) |
| Low | 322225 (55.5) | 206225 (54.2) |  |
| Moderate | 141938 (24.4) | 111605(29.3) |  |
| High | 116594 (20.1) | 62737 (16.5) |  |
| BMI (mean (SD), kg/m^2^) | 22.07 (2.45) | 22.09 (2.44) | 90986 (15.67) |
| NDVI (mean (SD)) | 0.21 (0.04) | 0.21 (0.04) | 0 (0) |

Abbreviations: SD, standard deviation; BMI, body mass index; NDVI, normalized difference vegetation index;

Table S2. Sensitivity analyses for the HRs (95% CIs) of respiratory mortality risk associated with each 1 µg/m^3^ in long-term particulate matters exposure

|  | PM_1_ |  | PM_2.5_ |  | PM_10_ |  |
| --- | --- | --- | --- | --- | --- | --- |
| Sensitivity analyses | HR (95% CI) | *P* value | HR (95% CI) | *P* value | HR (95% CI) | *P* value |
| Three different IPW methods | |  |  |  |  |  |
| Weighting by GPS using a LM model | 1.074 (1.063-  1.084) | <0.001 | 1.045 (1.040-1.051) | <0.001 | 1.044 (1.039-1.049) | <0.001 |
| Weighting by GPS using a ML model | 1.064 (1.054-  1.074) | <0.001 | 1.040 (1.035-1.046) | <0.001 | 1.040 (1.035-1.045) | <0.001 |
| Weighting by GPS using a GEE model | 1.066 (1.056-  1.076) | <0.001 | 1.042 (1.036-1.047) | <0.001 | 1.040 (1.036-1.045) | <0.001 |
| Restricted to |  |  |  |  |  |  |
| Complete case dataset | 1.091 (1.078-1.104) | <0.001 | 1.052 (1.046-1.060) |  | 1.042 (1.035-1.049) |  |
| Different NDVI exposures | |  |  |  |  |  |
| NDVI buffer 250m | 1.065 (1.055-1.075) | <0.001 | 1.041 (1.035-1.046) | <0.001 | 1.039 (1.035-1.044) | <0.001 |
| NDVI buffer 1000m | 1.065 (1.055-1.075) | <0.001 | 1.040 (1.035-1.046) | <0.001 | 1.039 (1.035-1.044) | <0.001 |

Abbreviations: HR, hazard ratio; CI, confidence interval; PM_1_, particulate matter with an aerodynamic diameter ≤ 1 µm; PM_2.5_, particulate matter with an aerodynamic diameter ≤ 2.5 µm; PM_10_, particulate matter with an aerodynamic diameter ≤ 10 µm; IPW, inverse probability weight; GPS, generalized propensity score; LM, linear model; ML, machine learning; GEE, generalized estimating equation; NDVI, normalized difference vegetation index;

Table S3. E-value for point estimates and the lower bound of the 95% confidence intervals of the hazard ratios^*^

| Models | PM_1_ | PM_2.5_ | PM_10_ |
| --- | --- | --- | --- |
| Weighting by GPS using a LM model | 1.355 (1.323) | 1.263 (1.243) | 1.259 (1.241) |
| Weighting by GPS using a ML model | 1.324 (1.291) | 1.245 (1.224) | 1.244 (1.225) |
| Weighting by GPS using a GEE model | 1.330 (1.299) | 1.250 (1.229) | 1.245 (1.228) |

^*^These estimated hazard ratios are obtained using three causal inference approaches

Abbreviations: HR, hazard ratio; CI, confidence interval; PM_1_, particulate matter with an aerodynamic diameter ≤ 1 µm; PM_2.5_, particulate matter with an aerodynamic diameter ≤ 2.5 µm; PM_10_, particulate matter with an aerodynamic diameter ≤ 10 µm; GPS, generalized propensity scores; LM, linear model; GEE, generalized estimating equation; ML, machine learning.


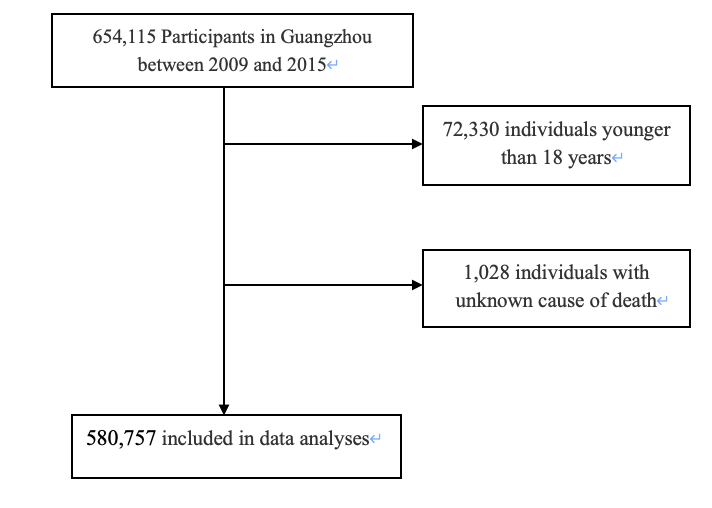


Figure S1. Flow chart of the study sample selection.


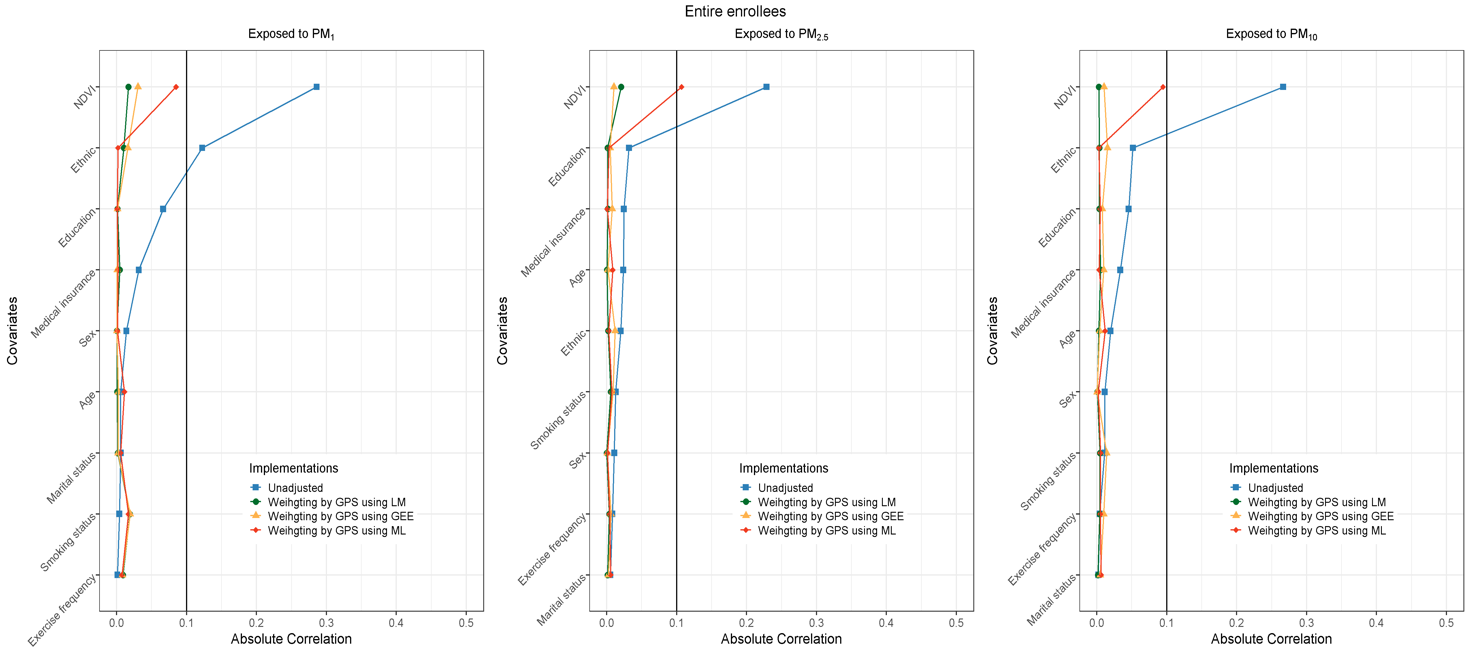


Figure S2. Mean absolute correlation for unadjusted, and weighted populations.

Abbreviations: PM_1_, particulate matter with an aerodynamic diameter ≤ 1 um; PM_2.5_, particulate matter with an aerodynamic diameter ≤ 2.5 µm; PM_10_, particulate matter with an aerodynamic diameter ≤ 10 µm; NDVI, normalized difference vegetation index; GPS, generalized propensity scores; LM, linear model; GEE, generalized estimating equation; ML, machine learning.


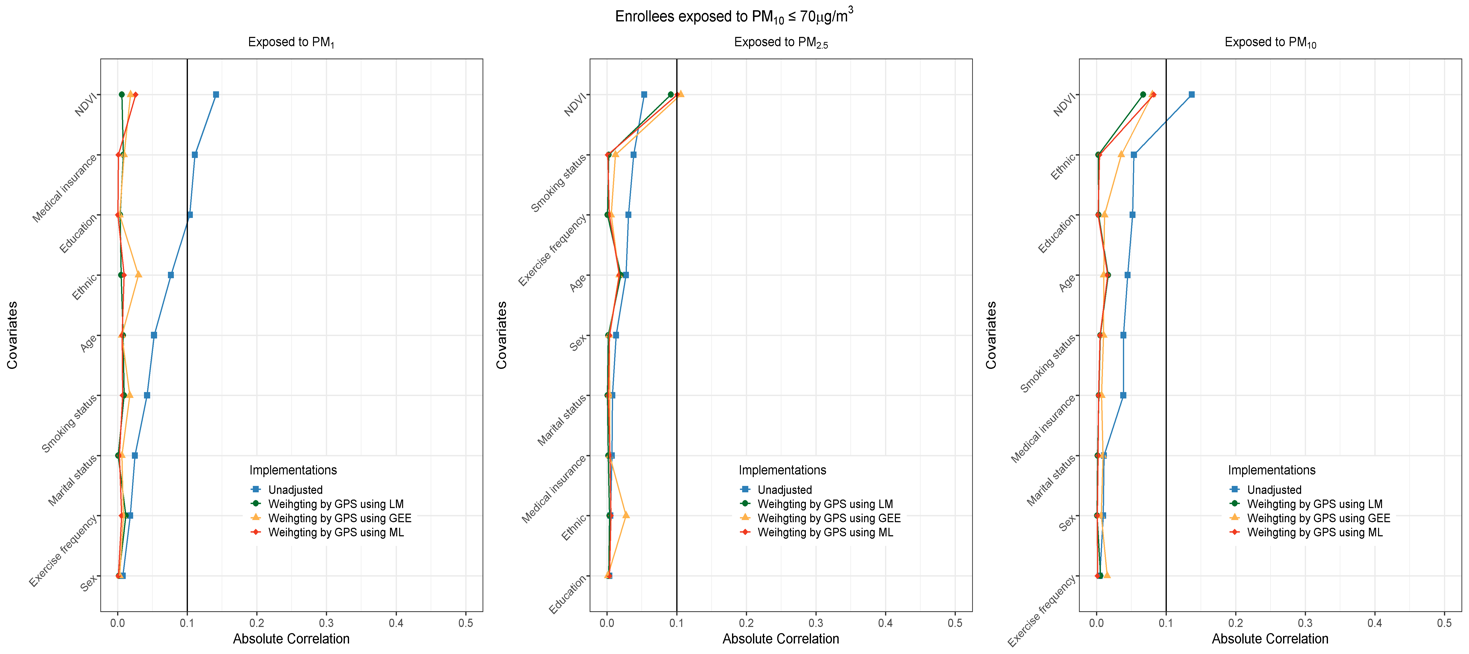


Figure S3. Mean absolute correlation for unadjusted, and weighted participants who always exposed to PM_10_ lower than 70 µg/m^3^.

Abbreviations: PM_1_, particulate matter with an aerodynamic diameter ≤ 1 μm; PM_2.5_, particulate matter with an aerodynamic diameter ≤ 2.5 µm; PM_10_, particulate matter with an aerodynamic diameter ≤ 10 µm; NDVI, normalized difference vegetation index; GPS, generalized propensity scores; LM, linear model; GEE, generalized estimating equation; ML, machine learning.


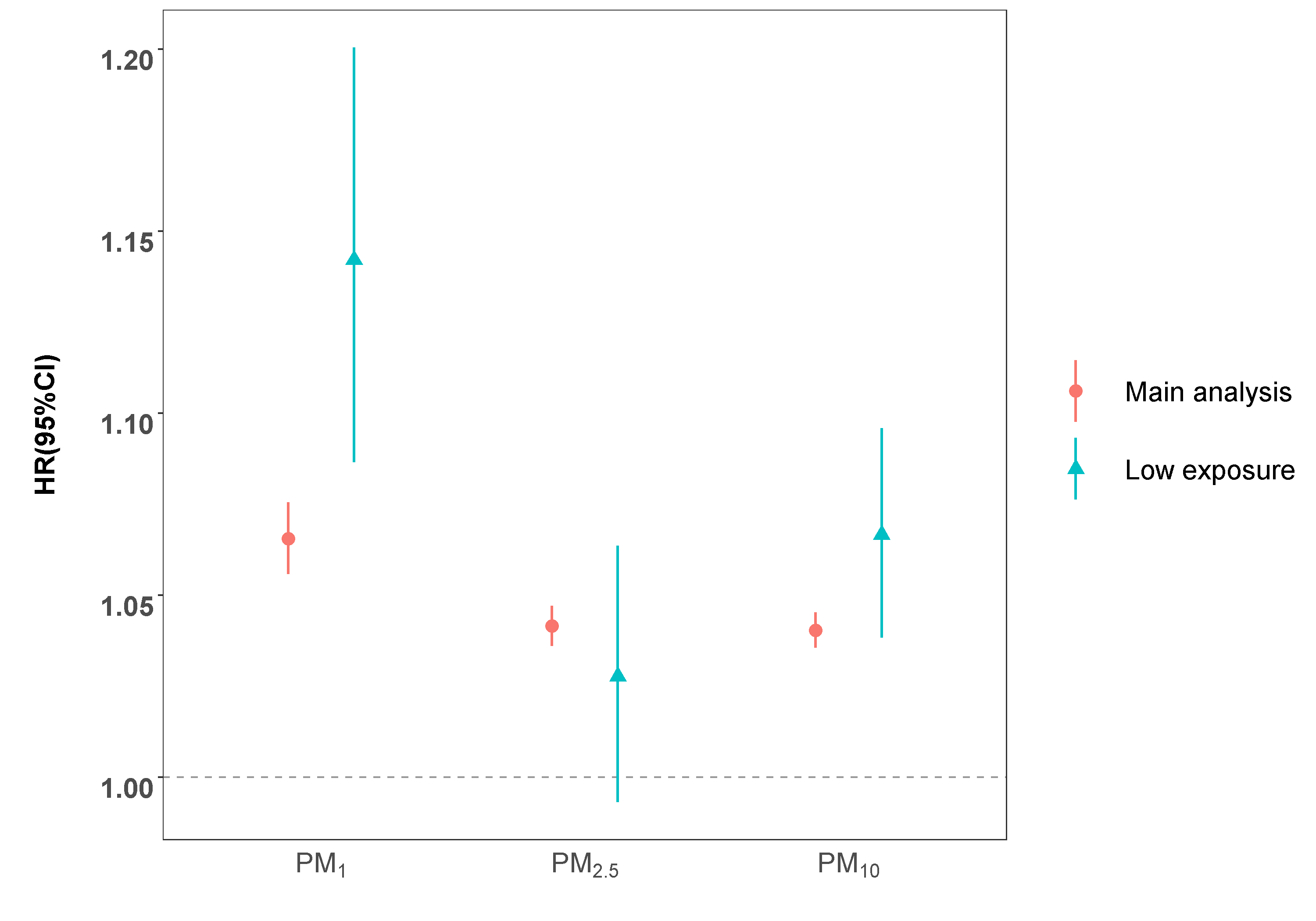


Figure S4. HRs (95% CIs) of respiratory mortality risk associated with each 1 µg/m^3^ in long-term particulate matters exposure among all participants and participants who were always exposed to PM_10_ lower than 70 µg/m^3^.

Note: adjusted for sex, ethnicity, education, marital status, medical insurance, exercise frequency and normalized difference vegetation index (500m).

Abbreviations: HR, hazard ratio; CI, confidence interval; PM, particulate matter; PM_2.5_, particulate matter with an aerodynamic diameter ≤ 2.5 μm; PM_10_, particulate matter with an aerodynamic diameter ≤10 µm; PM_10-2.5_, particulate matter with an aerodynamic diameter between 2.5 and 10 μm.
